# Evaluating the Potential and Pitfalls of AI-Powered Conversational Agents as Humanlike Virtual Health Carers in the Remote Management of Noncommunicable Diseases: Scoping Review

**DOI:** 10.2196/56114

**Published:** 2024-07-16

**Authors:** Sadia Azmin Anisha, Arkendu Sen, Chris Bain

**Affiliations:** 1 Jeffrey Cheah School of Medicine & Health Sciences Monash University Malaysia Bandar Sunway Malaysia; 2 Faculty of Information Technology Data Future Institutes Monash University Clayton Australia

**Keywords:** conversational agents, artificial intelligence, noncommunicable disease, self-management, remote monitoring, mobile phone

## Abstract

**Background:**

The rising prevalence of noncommunicable diseases (NCDs) worldwide and the high recent mortality rates (74.4%) associated with them, especially in low- and middle-income countries, is causing a substantial global burden of disease, necessitating innovative and sustainable long-term care solutions.

**Objective:**

This scoping review aims to investigate the impact of artificial intelligence (AI)–based conversational agents (CAs)—including chatbots, voicebots, and anthropomorphic digital avatars—as human-like health caregivers in the remote management of NCDs as well as identify critical areas for future research and provide insights into how these technologies might be used effectively in health care to personalize NCD management strategies.

**Methods:**

A broad literature search was conducted in July 2023 in 6 electronic databases—Ovid MEDLINE, Embase, PsycINFO, PubMed, CINAHL, and Web of Science—using the search terms “conversational agents,” “artificial intelligence,” and “noncommunicable diseases,” including their associated synonyms. We also manually searched gray literature using sources such as ProQuest Central, ResearchGate, ACM Digital Library, and Google Scholar. We included empirical studies published in English from January 2010 to July 2023 focusing solely on health care–oriented applications of CAs used for remote management of NCDs. The narrative synthesis approach was used to collate and summarize the relevant information extracted from the included studies.

**Results:**

The literature search yielded a total of 43 studies that matched the inclusion criteria. Our review unveiled four significant findings: (1) higher user acceptance and compliance with anthropomorphic and avatar-based CAs for remote care; (2) an existing gap in the development of personalized, empathetic, and contextually aware CAs for effective emotional and social interaction with users, along with limited consideration of ethical concerns such as data privacy and patient safety; (3) inadequate evidence of the efficacy of CAs in NCD self-management despite a moderate to high level of optimism among health care professionals regarding CAs’ potential in remote health care; and (4) CAs primarily being used for supporting nonpharmacological interventions such as behavioral or lifestyle modifications and patient education for the self-management of NCDs.

**Conclusions:**

This review makes a unique contribution to the field by not only providing a quantifiable impact analysis but also identifying the areas requiring imminent scholarly attention for the ethical, empathetic, and efficacious implementation of AI in NCD care. This serves as an academic cornerstone for future research in AI-assisted health care for NCD management.

**Trial Registration:**

Open Science Framework; https://doi.org/10.17605/OSF.IO/GU5PX

## Introduction

### Burden of Noncommunicable Diseases

Noncommunicable diseases (NCDs), also known as chronic diseases, are medical conditions that are not primarily caused by infectious agents (eg, viruses, bacteria, fungi, or parasites) and cannot be transmitted from one individual to another through close contact [1]. NCDs, such as cancer, cardiovascular diseases (CVDs), chronic obstructive pulmonary diseases, chronic respiratory diseases, chronic kidney diseases, cognitive disorders, metabolic syndrome, diabetes, and hypertension, are rising worldwide, with significantly higher rates in low- and middle-income countries (LMICs) [2-4]. In 2019, NCDs contributed to the highest proportion of total mortality (74.4%), accounting for 7.1 million additional deaths in 2019 as compared to 2009 [5]. Nearly half of the mortalities in Asia are attributable to NCDs, causing >80% of CVD and diabetes deaths, 90% of chronic obstructive pulmonary disease deaths, and two-thirds of all cancer deaths occurring in LMICs, resulting in 47% of the global burden of disease [3,6]. Major risk factors of NCDs include unhealthy dietary habits; physical inactivity; stress; and consumption of drugs, tobacco, and alcohol, which are generally modifiable due to lifestyle choices [5,7]. These chronic diseases are also major causes of long-term disabilities and prolonged costly treatment that may pose serious threats to a country’s health care resources and expenditure, especially among lower-income countries where health systems are not sufficiently equipped to tackle the escalating challenges [3,4,8].

In addition to regular visits to health care centers, NCDs require challenging self-care management, including compliance with medications, lifestyle modifications, and constant symptom monitoring to prevent disease progression; nonetheless, adherence to these procedures is generally low, especially among adults with limited health literacy who struggle to comprehend and follow instructions from their health care professionals [9,10]. Moreover, the shortage of health care providers and their limited time availability are substantial causes of patients’ being deprived of receiving adequate health education and support to make informed decisions required for the effective self-management of their chronic illnesses [11,12]. In addition, access to proper health services can be limited in many underdeveloped areas and rural communities due to poor health care infrastructure and mobility facilities [13].

The optimal prevention management strategy should incorporate elements of individual lifestyle management, societal health awareness management, national health policy decisions, and global health strategy [7]. However, the escalating prevalence of NCDs worldwide reveals that traditional disease management techniques are not sufficiently effective, thereby indicating an urgent necessity to develop effective supplementary management strategies to mitigate the substantial financial burden imposed by NCDs on many households, particularly in LMICs [2,3,14].

### The Role of Telehealth in NCD Management

Telehealth applications have the potential to improve patient self-care and disease-specific knowledge as well as minimize hospitalizations and mortality [15]. There is evidence from several past studies suggesting the significant effectiveness of mobile-based telehealth apps in improving nutritional intake and physical activity with technology intervention, resulting in body weight loss and adoption of recommended lifestyle changes due to their convenience and accessibility [16]. This is because such apps, with the help of existing and emerging technologies, have the ability to assist patients in managing their chronic diseases more effectively by providing constant self-monitoring tools and promoting improved self-management of health problems [17]. Furthermore, the ever-growing ownership of mobile devices worldwide has greatly contributed to the shift toward digital health care services, including assessment, monitoring, and treatment of physical and mental health, thereby indicating the promising ability of mobile health apps in self-monitoring, assessment, and treatment of NCDs at a reduced cost [18-20]. Indeed, the COVID-19 pandemic has expedited the use of telehealth [21,22], which has been proposed as a cost-effective method of delivering better health care services to people with chronic illnesses in a more flexible, personalized, transparent, dynamic, and accessible way [21,23].

### Potential Enhancement of Existing Telehealth Apps With Artificial Intelligence

Although mobile-based telehealth apps offer an ideal platform for systems designed to help patients manage their chronic illnesses due to the smartphones’ computational power, connectivity, and consistent availability, many individuals struggle with complex user interfaces of existing digital health technologies [10]. While the ubiquity of these apps reduces some acceptance barriers, most apps still overlook many other barriers, such as lack of motivational, psychological, and emotional support [10,24]. Nonetheless, technological advancement involving artificial intelligence (AI) seems to have the potential to further upgrade existing mobile health apps with more user-friendly features that can support individual user needs [25]. For instance, many patients lack the ability to effectively navigate conventional telehealth apps due to limited health-related or computer literacy and disabilities such as visual impairment; hence, such intelligent dialogue systems, specifically termed *conversational agents* (CAs), may help overcome these limitations and improve usability by providing an oral presentation of the apps’ contents in plain language [15]. AI-powered CAs are computer systems that can communicate with humans through text, voice, and images on mobile, web-based, or audio-based platforms using AI techniques such as machine learning (ML; a statistical method of training models using data for making predictions based on a variety of features) and natural language processing (machines’ ability to detect and interpret humans’ verbal and written languages) [26,27]. A CA may serve as an *empathetic* listener to understand patients’ problems as well as aid in monitoring a patient’s health 24/7 and notify physicians about an anticipated medical emergency [13].

The popularity of CAs, especially those that use unrestrained natural language, has increased over the last decade as consumers can use their smartphones to interact with CAs for daily tasks [27]. When deployed on mobile devices, CAs have the potential to augment human intelligence and demonstrate multiple benefits such as delivering health education and behavior change for a range of chronic health conditions [10]. Moreover, these AI-based CAs provide additional communication channels that are particularly effective for developing trust and therapeutic alliance to encourage adherence among users [10]. The human-like conversational features of CAs due to advancements in natural language processing, voice recognition, and AI are increasingly substituting human employees in service encounters, including the health care industry to deliver personalized care and support to individuals with chronic health issues [28].

### Types of CAs

In this paper, we broadly categorize intelligent CAs into 3 main types (Table 1): *chatbots* (text based or voice enabled—*voicebots*) without embodiment, computer-based embodied *digital avatars*, and physically embodied *humanoid robots* [29]. Although *robots* are beyond the scope of this paper, AI humanoid or social robots can also be classified as CAs that can be used as human-like health caregivers for managing NCDs. Despite the differences, all CAs, including humanoid robots, aim to enhance relational outcomes through human-like communication [29].

**Table 1:** Types of conversational agents (CAs).

AI^a^-based CA type (with examples)	Features and functionality	Interaction mode	Advantages	Challenges
Chatbots (eg, ChatGPT)	Computer programs integrated into messaging platforms that interact with users via free text [30] Evolved from preprogrammed, fixed scripted responses to AI-powered versions enabling more human-like conversations [31-33]	Text based	Simple user interfaces Easy and cheaper to design and develop	Offers mostly chat-based interactions Lack of personalized emotional tones [34]
Voicebots (or voice assistants; eg, Amazon Alexa and Apple’s Siri)	Voice-enabled intelligent chatbots that interact with users and respond to speaker commands primarily through voice [35,36]	Voice based (or text+voice enabled)	Offers greater flexibility by allowing for hands-free conversations and multitasking [35] Beneficial for users with limited digital expertise (eg, users with typing inabilities)	Development of enhanced voice interfaces crucial for a more natural conversation [36] Require some level of emotion awareness (a significant component of voice conversations) [36] Prone to speech recognition errors [37]
Digital avatars (eg, Replika, Mitsuku, and Soul Machines)	AI-created anthropomorphic representations of real-world characters in a computer-simulated environment, partially automated in some actions and movements, emulating human behavior [38,39] Digital AI avatars can generate human-like interactions through a fusion of multimodal features [40,41] Digital avatars evolved from 2D cartoonlike characters to visually realistic and interactive human faces with 3D imaging, leading to the emergence of highly realistic avatars (“Digital humans”) [42] Companies such as Soul Machines use CGI^b^, AI, and NLP^c^ to create digital human prototypes in metaverse spaces [43] Digital humans can represent fictional characters or virtual replicas of real humans, requiring digital twin technology for data-driven personalized care [44-46]	Multimodal (text based, voice activated, and face-to-face)	Ability to mimic natural human interactions by delivering highly personalized responses [40,41] Capable of face-to-face conversations, displaying physical nonverbal behaviors (eg, facial expressions, hand gestures, nodding, and head and body postures) [47]	Technical implementation is more complex, time-consuming, and resource intensive [47] Real-time video interactions with digital AI avatars require high computing power and bandwidth connectivity [48] Risk of perceived uncanniness due to increased realism, posing ethical concerns [25,49]
Humanoid (or anthropomorphic or social) robots (eg, Sophia and Ameca)	Designed to mimic human-like characteristics both in behavior and physical appearance, including body structure and autonomous movement [50,51] Often used for education, entertainment, assistance, and personal care through various sensor channels such as hearing, sight, and touch [52] Similar to digital avatars, humanoid robots can exhibit human-like communicative behaviors, including social praise, head and torso movements, and nodding, to stimulate more natural conversations [29]	Physical embodiment	Capability to perform difficult or dangerous tasks, provide companionship, and participate in social interactions, especially in circumstances where human interaction is limited [50,53] Physical presence enables more distinct and natural interactions compared to chatbots or virtual avatars as HRI^d^ differs from traditional HCI^e^ by involving both linguistic and physical aspects [29,52]	Higher development cost relative to other CAs Lower ease of access for users compared to other CAs [52,54] Higher risk of ethical and safety concerns

^a^AI: artificial intelligence.

^b^CGI: computer-generated imagery.

^c^NLP: natural language processing.

^d^HRI: human-robot interaction.

^e^HCI: human-computer interaction.

### Ethical Concerns of AI Agents

While AI agents have the capability to provide constant health surveillance support, challenges and risks associated with using AI-based CAs in health care remain. These include ethical concerns regarding data collection and interpretability of results, patient safety risks, biases encoded in algorithms, and cybersecurity [55,56]. Furthermore, customers are often reluctant to engage with such AI-based CAs due to many factors such as trust, reliability, learning curve, usability, privacy, and data security that should be addressed in the design, deployment, and use of AI applications [12,29,57-59].

### State-of-the-Art Summary

Our study is novel regarding CAs as human-like digital agents for managing NCDs remotely. As AI-based CAs are a relatively new area, limited research has been conducted on applying these emerging technologies in health care. While existing research on CAs as virtual caregivers for NCDs serves as a seminal foundation, such research has other critical limitations. To illustrate, most studies have primarily focused on applications of CAs for mental health conditions, overlooking broader NCDs such as CVDs, metabolic syndrome, or diabetes [60,61]. A recent review [61] that focused on a different set of research questions from ours concluded the following: “A future chatbot could be tailored to metabolic syndrome specifically, targeting all the areas covered in the literature, which would be novel.” Our research looks specifically into this gap.

Furthermore, despite some favorable anecdotal evidence, the effectiveness of CAs in NCD management is seldom explored in large-scale trials, particularly in older adults, who have the highest risk of developing NCDs [62-64]. Some recent previous studies have conducted systematic reviews [27,65,66] or scoping reviews [61,62,67] on CAs in chronic disease management; however, to the best of our knowledge, no review has been conducted yet on the application of AI-based CAs as human-like digital agents for managing NCDs remotely. Such limitations and gaps as highlighted previously present both challenges and opportunities for research to advance our understanding of how CAs can contribute to managing NCDs.

### Aim

This scoping review aimed to provide an overview of the existing evidence and research on using assistive humanoid AI-based CAs in health and social care for managing NCDs. Our primary objective was to explore the impact of AI-based CAs, including embodied avatars, as human-like health carers for the self-management of chronic diseases. By examining the current literature on this topic, we hoped to identify key areas for future research and provide insights into how these technologies can be effectively used in health care and personalize NCD management strategies.

### Research Objectives

Our research objectives were as follows: (1) to explore the current state of research on the use of AI-based CAs as human-like virtual health carers for managing NCDs, (2) to identify the potential benefits and challenges associated with the use of these technologies in the health care field, (3) to explore the efficacy of AI-based CAs in the remote management of NCDs, (4) to discover the specific target users primarily studied, and (5) to provide recommendations for future research in this area.

### Research Questions

Our research questions were as follows: (1) what is the current state of research on using AI-based CAs as human-like health carers for managing NCDs? (2) What are the limitations or challenges associated with the use of these technologies in health care? (3) What are the potential benefits of using AI-based CAs in managing NCDs, and how can they be effectively used to improve health care delivery and reduce health care burden? (4) What is the efficacy of the CAs in the remote care of NCDs? (5) What are the frequently targeted user groups for such virtual agents (eg, specific age groups and individuals with special needs)?

## Methods

### Search Strategy

We followed the methodological frameworks proposed by Arksey and O’Malley [68] and Levac et al [69]. Initially, research objectives and questions were formulated, followed by a systematic literature search conducted on July 31, 2023. For primary searching, we used 6 electronic databases that are considered relevant to the research focus—Ovid MEDLINE, Embase, PsycINFO, PubMed, CINAHL, and Web of Science—applying the same set of keywords, such as “conversational agents,” “artificial intelligence,” and “noncommunicable diseases,” including their associated synonyms (as shown in Textbox 1). The Boolean operators “*” and “OR” were used to expand and ensure that different word combinations were included. The operator “AND” was used for combining the main search terms to identify articles focusing on AI-based CAs (as health carers) only applicable in the health care field, particularly for managing NCDs or chronic diseases.

Additional studies were identified by hand searching the reference lists of included studies and relevant review articles. Furthermore, a supplementary manual search was conducted to identify specific articles from diverse sources, including ProQuest Central, ResearchGate, ACM Digital Library, and Google Scholar. Some of these articles were identified through pilot hand searching and preselected as they were closely relevant but not retrieved from the selected databases used for primary searching, whereas others were discovered through manual searches targeting specific authors well known in this field. The search strategy underwent refinement following expert recommendations, including insights from a coauthor with digital health expertise, who advised excluding “robots” from the search terms, recognizing it as a distinct field. These methodological steps collectively ensured a comprehensive exploration of the relevant literature.

**Search query example.**

**Example**

- (“conversational agent*” OR “relational agent*” OR “virtual agent*” OR “dialogue agent*” OR “dialogue system*” OR “virtual assistant*” OR “chatbot*” OR “voice assistant*” OR “voicebot*” OR “voice-bot*” or “voice bot*” OR “humanoid *bot*” OR “social *bot*” OR “avatar*” OR “human-like avatar*” OR “anthropomorphic avatar*” OR “digital human*” OR “human digital twin*” OR “virtual human*”) AND (“intelligent” OR “artificial intelligence” OR “AI” OR “AI-based”) AND (“health” OR “healthcare” OR “caregiver” OR “self-management” OR “self-monitor*” OR “non-communicable disease*” OR “noncommunicable disease*” OR “chronic disease*”)

### Eligibility Criteria

Our scoping review used comprehensive inclusion and exclusion criteria to ensure the selection of pertinent studies. We did not impose any limitations based on gender or age groups in the selection of articles. The search scope was confined to scholarly articles in English published between January 2010 and July 2023, aligning with the substantial rise in CAs after 2010 [70], notably with the introduction of Apple’s Siri in 2011 [71]. Evidently, most of the selected papers were on recent studies conducted within the last 5 years due to the accelerated technological advancements and the latest evolution of AI. Specifically, we focused on empirical studies exploring CAs applied exclusively to human interaction within the health care context, emphasizing their role in the remote management of NCDs, ideally within home environments.

The exclusion criteria comprised the exclusion of conference abstracts, posters, reviews, protocols, position papers or viewpoints, and certain types of studies such as those involving noninteractive robotic devices. We also excluded studies related to CAs designed for medical education and non–patient-centered applications in hospital settings and those addressing specific health care domains such as surgery, dentistry, pregnancy or maternity, addiction or substance use disorders, and communicable diseases. Furthermore, we excluded studies centered solely on medical history data storage, telephone monitoring, data set construction methods, or user evaluations of commercial CAs for health care without their practical applications in the remote management of NCDs. Our criteria aimed to streamline the focus on patient-centered CAs contributing to the self-management and remote monitoring of NCDs, eliminating studies with a primary emphasis on clinical interviews, disease prediction, or decision support without a social interaction element.

### Screening and Selection

In total, 2 of the authors independently searched each database. Titles and abstracts were screened for inclusion according to the aforementioned criteria, followed by an exclusion of duplicates, unrelated studies, and articles that could not be retrieved. The abstract screening yielded 264 articles eligible for full-text screening, of which 70 (26.5%) were review papers comparing different types of CAs in the health care field and 156 (59.1%) were empirical studies that explored different types of CAs used in the prevention, treatment, or rehabilitation of chronic diseases involving consumers, caregivers, and health care professionals.

**Figure 1:**
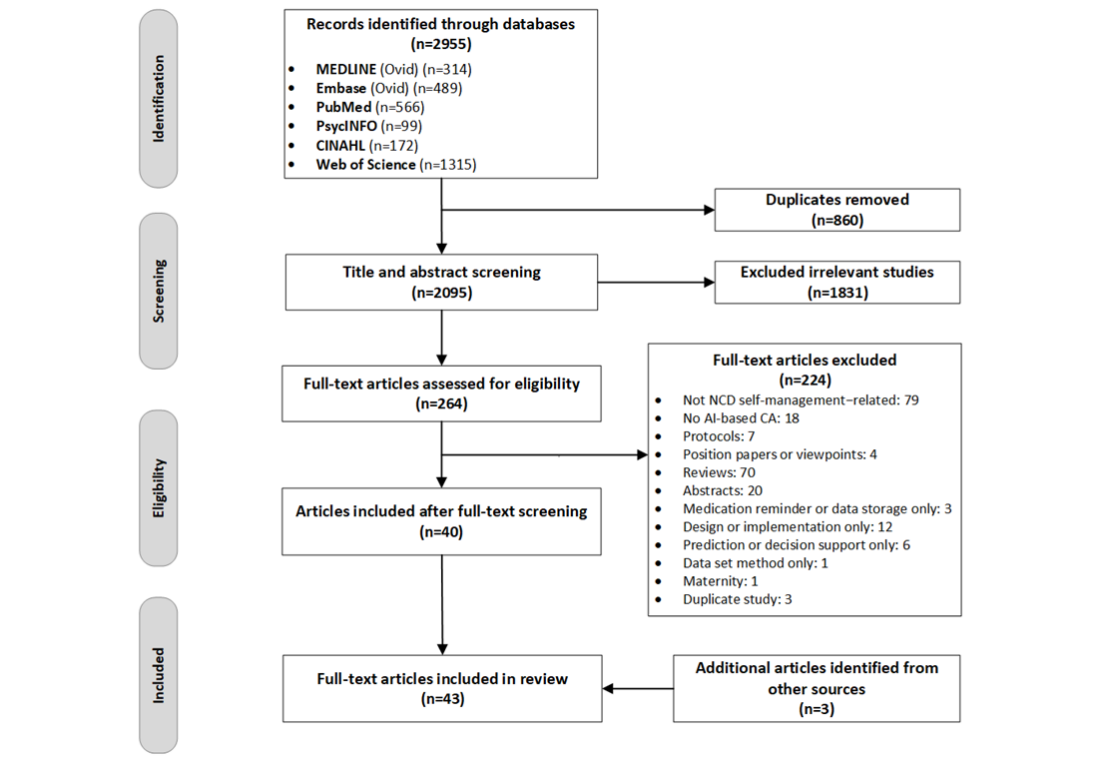
PRISMA (Preferred Reporting Items for Systematic Reviews and Meta-Analyses) flowchart of the search and screening process. AI: artificial intelligence; CA: conversational agent; NCD: noncommunicable disease.

Subsequently, the authors screened full texts of the remaining articles independently, and 40 full-text articles that met the inclusion criteria were selected for review. In addition, 10 [10] specific articles were obtained from hand searches of other sources (Google Scholar, ProQuest, ACM Digital Library, and ResearchGate), of which 3 (30%) [3] relevant ones were selected upon full-text screening. It was an iterative process; any discrepancies were discussed among the authors. The search and selection process is illustrated in Figure 1.

### Data Collation and Reporting

An Excel (Microsoft Corp) spreadsheet was initially created to aid the screening and selection process. Following the screening process, a total of 43 articles were eventually selected for synthesis. Quantitative and qualitative data from the included studies were extracted and summarized in a tabular format, including information such as intervention, type of CA, target population, number of participants with their age, methods, study duration, location, measures and outcomes, and limitations. The relevant extracted information was collated and summarized using the narrative synthesis approach, which was deemed appropriate for capturing the breadth of evidence in scoping reviews, identifying themes aligned with the research questions as well as patterns observed across the included studies and relevant reviews. The results were reported according to the PRISMA-ScR (Preferred Reporting Items for Systematic Reviews and Meta-Analyses extension for Scoping Reviews) guidelines. Given the divergent methodologies of the included studies, predominantly using mixed methods and qualitative approaches, no quantitative synthesis or meta-analysis was conducted.

## Results

### Characteristics of the Included Studies

Most of the included studies (22/43, 51%) were feasibility, acceptability, and usability studies (Table 2). These studies mostly found positive results in terms of feasibility and usability of the proposed CAs. Some studies (4/43, 9%) used the System Usability Scale questionnaire to measure the system’s usability score and predominantly found high usability, with System Usability Scale scores of >70 [72-75]. Alternatively, multiple studies (10/43, 23%) used the subjective ratings of most of the participants to evaluate the feasibility and acceptability of CAs by applying indicators such as perceived usefulness [76-79], ease of use [78,80], user satisfaction [80,81], engagement rate [80,82,83], perceived closeness [73], and Net Promoter Score [84]. However, 2% (1/43) of the studies reported negative usability and acceptability outcomes, indicating unsatisfactory reliability, usability, and goal structure, which hindered health care professionals’ acceptance and trust [85].

**Table 2:** Characteristics of the included studies.

Study, year	Intervention and study duration	CA^a^ type and delivery platform	Target population, sample size, and participants’ age	Methods and location	Measures and outcomes	Limitations
Watson et al [86], 2012	Behavior change intervention for obesity self-management Duration: 12 weeks (divided into four 3-week periods—period 1, period 2, period 3, and period 4)	2D-animated human-like avatar (rule-based AI^b^) Platform: desktop and laptop computers (web based)	Adults with overweight or obesity N=62 Participants aged 20-55 years (inclusive)	Pretest-posttest observational study (quantitative)—2-arm RCT^c^ Location: United States	Step count percentage: No significant percentage change in step count between intervention and control arms from start to end (2.9% vs −12.8%, respectively; *P*=.07) Significant difference between percentage change in step count across all study periods in the intervention vs control arms (*P*=.02) Secondary outcomes: no significant changes in secondary outcomes—BMI, 7-day physical activity recall, physical activity stage of change, self-efficacy and exercise benefits and barriers, and program satisfaction (eg, mean decrease in BMI of 0.04 and 0.25 in control and intervention groups, respectively; *P*=.44) Average step count: significant decrease in the average mean step count for the control group from period 1 (7174) to period 4 (6149; *P*=.01) but no significant change for the intervention group from period 1 (6943) to period 4 (7024; *P*=.85) Mean activity level: significant percentage change in mean activity levels between period 1 and period 3 (*P*=.02) but no significant change between period 1 and period 2 (*P*=.12) and between period 1 and period 4 (*P*=.07) 58% of the intervention participants agreed that the virtual coach influenced their increased activity	Participants were primarily White, college-educated women, limiting generalizability of findings to a wider patient population with overweight or obesity Lack of baseline step count data for participants, although survey results showed no substantial baseline activity level differences Initial observed increase in step count after enrollment likely reflects a change from baseline, but without baseline data, this cannot be confirmed
Kimani et al [80], 2016	Lifestyle intervention and patient counseling support for AF^d^ self-management Duration: not identified	Animated human-like avatar (network-based AI with XML scripting language) Platform: mobile devices or smartphones	Patients with AF N=16 (5 female and 11 male) Participants aged ≥18 years (20-58 years)	Feasibility assessment—mixed methods pilot study with self-report scale measures and a semistructured interview Location: not identified	High overall user satisfaction with the agent (mean 3.45 on a 4-point scale) and the AliveCor heart rhythm monitor (mean 3.54) High ratings for ease of use (mean 3.54) Interaction duration: participants reported a 7- to 10-minute–long interaction with the agent, whereas older participants reported longer interactions due to content relevancy Agent’s user satisfaction correlated with the participants’ satisfaction with the use of the AliveCor heart rhythm monitor Acceptable feasibility of delivering AF counseling via a smartphone-based humanoid avatar as a virtual agent	Small sample size and limited patient diversity Short-term study duration and no long-term use evaluation No control group for comparison to provide additional insights into the effectiveness of the virtual agent No assessment of the virtual agent’s impact on patient outcomes or behavior change
Shamekhi et al [87], 2017	Behavioral intervention for self-management of stress and depression Duration: 9-21 weeks	3D-animated human-like avatar (rule-based AI) Platform: touch-screen tablets	Adults with chronic pain and depression N=154 Participants aged≥18 years	Mixed methods observational study—2-arm RCT Location: United States	Impact on patient performance and satisfaction: significant positive stress management behaviors among the CA intervention participants compared to the control group after 9 weeks (t136=3.74; *P*<.001) Avatar (Gabby) was found to be very useful for group visits, allowing for review of class lessons and detailed information at any time and location Most participants found the meditation, yoga, and mindfulness sessions provided by Gabby to be very “useful” and “relaxing” Participants were very likely to recommend Gabby to others	Relatively small sample size from health centers in the Boston area, limiting generalizability of findings to other populations Inability to isolate the effects of face-to-face weekly group visits from those of the home-based CA Potential usability issues, especially for less computer-literate users; may affect Gabby’s user satisfaction
Bickmore et al [10], 2018	AF self-management through symptom monitoring and patient counseling Duration: 30 days	3D-animated human-like avatar (network-based scripted AI) Platform: mobile devices or smartphones	Older adult patients with AF N=120 Participants aged≥60 years (mean 72.1, SD 9.10 years)	Mixed methods pretest-posttest (observational) study—quasi-experimental demonstration of CA and 2-arm RCT for usability and effectiveness evaluation Location: United States	Change in AFEQT^e^ score: significantly higher AFEQT score among the intervention group participants after 30 days compared to those in the control group (*P*<.05) Over 89.7% of participants rated the CA positively, whereas 4.4% rated it negatively and 5.9% had mixed opinions Participants praised the personalized interaction with the avatar	Relatively small convenience samples with limited participant details and a singular focus on a specific chronic condition (AF) may impact generalizability Lack of long-term, objectively measured health outcomes Insufficient emphasis on the complexity of self-care management regimens, especially for patients with low health literacy
Cheng et al [58], 2018	Personalized patient education and medication adherence intervention for improved self-management of T2DM^f^ Duration: 1 month	AI voice assistant and voicebot (ML^g^ based) Platform: Google Home devices (API.AI platform)	Older adult patients with T2DM N=10 Participant age not identified	Feature-based comparison between the proposed voicebot and other similar mHealth^h^ apps and usability evaluation—qualitative assessment Location: not identified	AADE^i^ requirement evaluation: the feature-based comparison indicates that most similar mHealth apps fail to meet the AADE requirements for effective T2DM self-management compared to the proposed voicebot Practical usability evaluation: There were more satisfied users than unsatisfied ones, primarily due to the voicebot’s speaker functionality and natural conversation flow Unsatisfied users reported difficulty learning commands and limited answer choices 80% of older adult participants would prefer using Healthy Coping on Google Home over a smartphone	Voicebot’s limitations Narration speed concern: older adult users may struggle with application commands due to narration speed; a speed setting could allow users to adjust the pace Accessibility limitations: Healthy Coping’s voice interface may not fully support users with hearing or speech disabilities; integrating devices such as Bluetooth-enabled hearing aids could improve accessibility
Suganuma et al [88], 2018	Internet-based cognitive behavioral intervention for self-monitoring of mental health Duration: 1 month	AI (rule-based) chatbot via SABORI (mental health) app Platform: web based	Adults with psychological distress and mental health problems N=454 (intervention group: n=191; control group: n=263) Participants’ age: mean 38.07 (SD 10.75) years	Feasibility and acceptability (pilot) study—nonrandomized prospective study with quantitative pretest-posttest intervention questionnaire Location: Japan	Japanese version of the WHO-5^j^: a 1-factor scale to measure positive mental health based on physical aspects using a 5-item, 6-point Likert scale On the variable of positive mental health, a significant main effect (*P*=.02) and interaction for time (*P*=.02), including a significant simple main effect for the intervention group (*P*=.002) K10^k^: a 1-factor scale to measure negative mental health based on physical aspects using a 10-item, 5-point Likert scale On the variable of negative mental health, a significant main effect (*P*=.02) and interaction for time (*P*=.005), including a significant simple main effect for the intervention group (*P*=.001) BADS^l^: a scale to measure 4 factors-“Activation” (BADS-AC^m^), “Avoidance/Rumination” (BADS-AR^n^), “Work/School Impairment” (BADS-WS^o^), and “Social Impairment” (BADS-SI^p^) 2-way ANOVA test showed a significant trend for behavioral activation, indicating the possibility for a certain degree of effectiveness BADS-AC: a significant main effect for time (*P*=.10), including a significant simple main effect trend in the intervention group (*P*=.06) BADS-AR: no significant main or interaction effect	Nonrandomized comparison between control and experimental groups Short-term study duration (1 month) for assessing behavioral activation effects Recruitment of healthy participants in control group may have influenced outcomes, resulting in lack of effect for behavioral activation factors of avoidance and rumination
Hussain and Athula [89], 2018	To provide patient education for improved self-management of diabetes Duration: not identified	AI-ML chatbot (VDMS^q^) Platform: computer or mobile devices (web based)	Patients with diabetes and their carers seeking diabetes-related information N=10 Participants aged 20-50 years	Quantitative and qualitative study—performance evaluation of VDMS compared to other information sources (eg, search and websites) Location: not identified	Evaluation metrics (VDMS vs other sources): Correct replies: VDMS=65%; search engine=80% Satisfactory user satisfaction level of VDMS (very close to that of the search engine) due to VDMS’s timely response and correct answers’ quality and clarity	Small sample size (10 participants) may restrict diversity and evaluation scope VDMS chatbot’s implementation is incomplete and lacks full testing Conversation is mostly controlled by the chatbot, limiting user input Extracted data from Wikipedia may lack reliability as no published work has explored integrating Wikipedia knowledge with a diabetes management chatbot
Inkster et al [90], 2018	To deliver positive psychology and mental well-being techniques for improved self-management of depression and anxiety Duration: 8 weeks	Commercial CA: text-based AI chatbot (Wysa) Platform: mobile devices or smartphones	Individuals with self-reported symptoms of depression N=not identified Participants’ age not identified	Quasi-experimental (pretest-posttest) design—mixed methods study using the Wysa app’s inbuilt assessment questionnaire (for effectiveness evaluation) Location: global (not specified)	Impact (pre-post) analysis outcomes: PHQ-9^r^ score (within groups) measured using a Wilcoxon signed rank test: significant reduction in PHQ-9 score among both high users (*P*<.001) and low users (*P*=.01), indicating an improvement in depression symptoms from before to after the intervention High users’ group showed significantly higher average mood improvement compared with the low users’ group (Mann-Whitney *P*=.03, with a moderate effect size of 0.63) User engagement and feedback: 73.6% of users provided at least one response to the in-app feedback questions (indicating high user engagement), and 60.9% of them reported feeling better after app use	Small sample size restricts investigation of user reactions to app design elements Small and unbalanced comparison group sizes undermine the findings’ generalizability Limited detailed feedback on users’ app experience hinders qualitative analysis Lack of a randomized controlled environment may introduce biases Absence of users’ previous health information hampers comprehensive understanding Quasi-experimental design used, slightly lower in design quality compared to interrupted time-series designs
Neerincx et al [85], 2019	Behavioral intervention and patient education for self-management of T1DM^t^ Duration: 3 months	Humanoid robot (PAL^u^) and its robotlike digital avatar version for home (MyPal; rule based+ML with cloud computing) Platform (avatar): tablets	Children with T1DM N=49 Participants aged 7-14 years	Qualitative study—prototype design and usability evaluation using an iterative, incremental development process Location: Italy and the Netherlands	Unsatisfactory reliability, usability, and goal structure of PAL system, which hindered the acceptance and trust of health care professionals Improvement in children’s diabetes knowledge when using the PAL system and increased motivation in performing diabetes-related activities due to enjoyable interaction with the PAL robot and avatar Improvement suggestions: personalization enhancement required to establish patients’ adherence Innovative PAL functions (eg, experience-sharing function) were identified and tested with positive results Reduced bonding effect with higher perceived similarity between the robot and its digital avatar Most children stopped using the avatar (MyPAL app) some weeks after the study started	Small sample size may affect the findings’ generalizability Limited study duration may not capture the PAL system’s long-term effects on T1DM management Comparison between physical robot and avatar function may introduce biases in user preference assessment Insufficient detailed information on AI algorithms or ML techniques used restricts insights into the PAL system’s technical aspects
Easton et al [91], 2019	Self-management for people with comorbid long-term conditions and mental health problems Duration: not identified	AI chatbot with an animated human-like 2D avatar (Avachat) Platform: computers, tablets, smartphones, and televisions	Older adults with comorbid long-term conditions and mental health problems N=10 Participants aged ≥55 years (56-86 years)	Co-design and acceptability testing involving stakeholders—qualitative study with snowball sampling and workshops for initial user requirement gathering of prototype and user feedback Location: United Kingdom	Acceptability and feasibility: Patients found Avachat to be helpful, informative, and easy to use Health care professionals are optimistic about the avatar’s potential to improve patient outcomes and reduce the health care burden Improvement suggestions: enhance personalization of contents and ensure accessibility for users with visual and hearing impairments	Small sample of White, British, medically stable, and regional participants recruited may limit findings No current mental health problems reported by participants, although past instances of low mood or worry were mentioned
Chaix et al [92], 2019	To provide personalized patient education for improved quality of life and medication adherence in patients with breast cancer Duration: 1 year	Commercial CA—AI chatbot (Vik) Platform: mobile devices or smartphones (iOS or Android) and web browser via Messenger app	Patients with breast cancer and their relatives N=4737 Participants’ age not identified	Quantitative study using user-chatbot conversational data Location: not identified	Users’ interactivity level: average of 132,970 messages exchanged per month, with 147 total average interactions per question (for open-ended questions), resulting in 2.7 interactions per person per question Overall user satisfaction rate: 93.9% (900/958), and 88% (843/958) found Vik helpful in following treatment effectively Average medication adherence rate of patients improved by >20% (*P*=.04) after using Vik for 4 weeks	Absence of a control group for comparison Lack of long-term evaluation of the chatbot’s impact on clinical outcomes Reliance on users’ self-reported data, introducing potential bias No investigation of the viewpoints of health care providers or other stakeholders in patient care Potential technical issues or limitations of the chatbot not addressed
Stephens et al [76], 2019	Behavioral intervention for constant management of obesity and prediabetes symptoms Duration: 6 months	Commercial CA—AI behavioral coaching chatbot (Tess) Platform: mobile devices or smartphones via messaging apps (eg, WhatsApp and Messenger); Google Home or Amazon Alexa (for voice conversations)	Adolescent pediatric patients with obesity and prediabetes symptoms N=23 Participants aged 9-19 years	Feasibility study—mixed methods approach with qualitative interviews, usability testing, and quantitative surveys (SUS^v^ questionnaire) Location: United States	Positive progress toward goals was reported by the participants 81% of the time 4123 messages were exchanged, with patients rating the chatbot’s usefulness 96% of the time, indicating its high perceived usefulness and feasibility among adolescents	Small sample size, causing limited generalizability Gradual program adjustments may introduce variability and potential inconsistency Lack of an experimental design to control for factors Inability to ensure detection of a treatment effect
Balsa et al [72], 2020	Behavioral intervention for self-management of T2DM Duration: 8-10 days	Animated human-like avatar (Vitória) in a 3D environment (rule-based AI) Platform: mobile devices or smartphones	Older adults with T2DM N=20 Participants aged ≥65 years (67-80 years)	Usability study—qualitative assessment Location: Portugal	Usability evaluation for T2DM medication adherence and lifestyle improvement Usability (SUS) score: 73.75 (SD 13.31; indicates significantly high usability of Vitória)	Small sample size and skewed participant sample toward expert technology users Reliance on previous experience and available resources for sample size estimation Criticism of questionnaires for yielding only overall measures without addressing specific concerns Potential bias in field-testing compared to laboratory setting for usability tests
Gong et al [24], 2020	Behavioral intervention with personalized support and motivational coaching for remote self-management of T2DM Duration: 12 months	Human-like (text+voice enabled) avatar (Laura; BCT^w^-based prescripted AI) Platform: mobile devices or smartphones	Adults with T2DM N=187 Participants aged ≥18 years (mean 57, SD 10 years)	Pretest-posttest (quantitative) observational study—2-arm, open-label RCT for adoption, usefulness, and effectiveness evaluation Location: Australia	Program adoption and use: Number of valid chats with Laura completed per person: 18.4 (SD 15.0; range 1-53) Total duration of valid chats per person: 242.7 (SD 212.3; range 0-1050) minutes Number of glucose level uploads per person: 181.8 (SD 192.1; range 1-966) Number of clinical alerts: total=297; average per month=13.7 (SD 8.8) Number of technical alerts: total=179; average per month=8.3 (SD 6.5) Number of posts on the web-based discussion forum: total=19; average per month=1.1 Program effectiveness (in terms of coprimary and secondary outcomes)—coprimary outcomes: Statistically significant between-arm difference at 12 months in the mean change in HRQoL^x^ (AQoL-8D^y^ utility value: 0.04, 95% CI 0.00-0.07; *P*=.04) Reduction in HbA_1c_^z^ levels during the trial but no statistically significant between-arm difference at 6 months (0.06, 95% CI −0.35 to 0.47; *P*=.78) or 12 months (−0.04, 95% CI −0.45 to 0.36; *P*=.84) Significant improvement in HRQoL from baseline to 12 months (mean estimated change in AQoL-8D score: 0.04, 95% CI 0.01-0.06; *P*=.007) Increase in the score of the physical health and mental health subscales compared with baseline Secondary outcomes: Significant between-arm difference in the mean change in the HADS^aa^ anxiety score at 6 months (–0.89, 95% CI –1.74 to –0.04; *P*=.04) but not at 12 months or for other secondary outcomes reported	Small sample size may restrict the generalizability of the findings, and subgroup analyses require cautious interpretation Absence of blinding of participants and their GPs^ab^ to the study arm allocation might lead to potential self-report bias and Hawthorne effects Control arm participants showed a higher rate of completed assessments, possibly due to their interest in program access or higher attrition in the intervention arm Subgroup analyses were underpowered, with multiple testing increasing the risk of false positives
Issom et al [77], 2020	Health behavioral intervention for self-management of SCD^ac^ Duration: not identified	AI chatbot Platform: mobile devices or smartphones	People with SCD N=19 Participants’ age not specified	Preliminary feasibility study—quantitative posttest survey Location: France	88% of participants rated the following question—“The chatbot contains all the information I need”—with at least 3/4, and its total score was 54/68 58% rated the following question—“The chatbot encouraged me to be more active in order to improve my condition”—with at least 3/4, and its total score was 51/68 Results indicate high perceived usefulness of the chatbot in promoting knowledge and motivation for improved self-care practices	Small sample size: only 17 participants completed the evaluation, potentially limiting generalizability 2 withdrawals due to smartphone issues may have impacted data collection and results Reliance on self-reported data in the posttest survey could introduce bias or inaccuracies Limited scope: evaluation focused solely on perceived usefulness of the chatbot, lacking assessment of long-term impacts on self-care or health outcomes Lack of comparison with other support forms or interventions hinders assessment of relative effectiveness
Anastasiadou et al [93], 2020	To offer continuous health education and interaction in English, Spanish, and Bulgarian for self-management of diabetes Duration: 6 months	Multilingual AI-ML chatbot (EVA^ad^) Platform: mobile devices or smartphones	People diagnosed with diabetes N=not identified Participants’ age not identified	Qualitative pilot study—validation and acceptance evaluation by integrating EVA into an mHealth app (CHRODIS PLUS Joint Action) to collect data on user queries and responses provided based on the educational content Location: Greece	Users sent a total of 940 unique messages to EVA Users’ common questions related to diabetes varied based on EVA’s language: English: self-measurement and understanding what diabetes is Spanish: glucose self-monitoring Bulgarian: insulin and high blood pressure A comprehensive analysis of the effectiveness of EVA in improving diabetes management or patient outcomes was not provided	Lack of detailed user demographic information may affect generalizability Study duration limited to 6 months, possibly overlooking long-term user interactions and feedback Absence of specified measures to address potential biases in user interactions or data collection No discussion about potential technical limitations ad challenges during EVA system development and testing
Roca et al [94], 2021	Medication adherence intervention for improved self-management of T2DM and depressive disorder Duration: 9 months	AI-ML–based chatbot Platform: mobile devices or smartphones	People with comorbid T2DM N=13 Participants aged ≥18 years	Quantitative pilot trial—pretest-posttest observational study (1 arm) Location: Spain	Medical outcomes: Significant improvement in the average HbA1c level and PHQ-9 scores from 7.6 (SD 0.7) to 7.3 (SD 0.8) and from 13.2 (SD 6.0) to 8.6 (3.6), respectively Reduction in the number of physical medical consultations per month from 2.0 (SD 2.6) to 1.3 (SD 1.5) in 30.8% of the patients Health care professionals participating in the study found the chatbot useful in improving medication adherence Chatbot feedback: Participants who used the chatbot daily found it useful in fulfilling their medication reminder needs The chatbot’s language and vocabulary were appropriate and easy to understand 38% of participants had difficulty learning to use the chatbot, and 15.4% reported the chatbot’s inability to understand the users’ instructions Almost 70% of the patients (9/13) expressed willingness to continue using the chatbot after the study	Small sample size (13 participants) may limit generalizability of the study findings Some patients did not update medication information after the initial configuration, leading to reminders being stopped Patients require digital literacy or assistance for configuring the messaging platform Reminder sounds may be accidentally disabled by patients, affecting the use of the chatbot
Yao et al [95], 2021	Remote monitoring of patients after stroke and automation of suspected stroke screening Duration: not identified	Hyperrealistic human-like 3D avatar (iLAMA; ML based with computer vision algorithms) Platform: mobile devices or smartphones and tablet	Older adult patients after stroke N=140 (videos) Participants aged ≥60 years	Quantitative beta testing of the prototype on 140 videos with volunteers via email Location: not identified	The app was able to recognize body parts and extract 68 facial landmarks from the facial videos The app could provide accurate stroke screening results for neurologists and stroke specialists to review No major technical issues or problems with the app during beta testing were reported iLAMA has the potential to improve stroke assessment and care, particularly in areas with limited access to stroke specialists	Participants were regular volunteers (friends and family) rather than patients after stroke Beta testing involved a relatively small sample size of 140 videos The app may struggle to differentiate minor mistakes from actual stroke signs, especially without supervision Screening process takes approximately 5 minutes, which some users may find burdensome
Krishnakumar et al [82], 2021	Lifestyle intervention and self-monitoring of diet, exercise, weight, and blood glucose for T2DM management Duration: 16 weeks	AI chatbot via mHealth therapeutic app (Wellthy Care) Platform: Android smartphones	Adults with T2DM possessing Android smartphones N=102 Participants aged ≥18 years	Retrospective study design—pretest-posttest (quantitative) observational study for effectiveness evaluation Location: India	Primary outcomes: Statistically significant reduction in HbA1c levels from baseline to 16 weeks (*P*<.001) Secondary outcomes: Mean FBG^ae^ and PPBG^af^ levels decreased significantly from baseline to 16 weeks (*P*<.001) Mean BMI and weight decreased significantly from baseline to 16 weeks (*P*<.001) Average duration of interactions was 106 minutes with the app (87 participants; 95% CI 65-147 minutes) and 88 minutes with the AI chatbot (102 participants; 95% CI 66-110), indicating a significant positive association with program engagement	Short program duration No control group as real-world data were retrospectively analyzed Selection bias from multiple selection approaches (physician recommended and voluntary approach) of participants Reliance on self-reported disease biomarkers Loss of data during follow-up Variation in the number of male and female participants, introducing potential bias
Egede et al [96], 2021	To improve mental health (depression and anxiety) assessments and remote counseling via VH^ag^ mediation Duration: not identified	Human-like avatar (Greta; Wizard-of-Oz AI system using prescripted text and human-controlled responses) Platform: not identified	Adults with anxiety and depression N=56 Participants aged ≥18 years (18-45 years)	Mixed methods comparison and UX^ah^ evaluation study: Behavioral expressiveness comparison between VH system and text-only system users UX assessment and task ratings using UEQ^ai^ and TAM^aj^ questionnaires Location: Japan	Stronger visual cues produced by VH avatar guidance compared to non-VH (*P*=.04; 0.036 for 2 facial action units indicating a statistically significant difference in the frequency of activation of these facial action units between the VH and non-VH systems; *P*=.005 and 0.0032 for 2 head pose descriptors indicating a statistically significant difference in head pose characteristics between the VH and non-VH systems) VH users make wider and broader head movements (indicating higher behavior expressiveness) compared to text-only users (*P*=.005 and 0.0032 for 2 head pose descriptors) VH mediation improves users’ inclination toward tasks, as indicated by the UEQ and TAM questionnaires Significant differences (*P*=.05) found between the system modes in task ratings for facial expression mimicking (*P*=.04), emotion recall (*P*=.01), thematic apperception (*P*=.03), and mindfulness (*P*=.04) tasks, with VH mode’s mean ratings significantly higher than those for the text-only mode except for the emotion recall task, where VH’s ratings were lower Groups with moderate depression exhibit more visible activity compared to groups with less depression (*P*=.04 and 0.004 for 2 facial action units)	Small sample size may limit depth of analysis, particularly in comparing depression severity classes Combination of moderate and moderately severe depression groups may restrict findings; a 4-class comparison could yield more valuable data Focus on VH mediation may have overshadowed other factors influencing user engagement and task performance Long-term effects of VH-mediated tasks on user engagement and mental health outcomes were unexplored, limiting insights into intervention sustainability
Romanovskyi et al [97], 2021	Cognitive behavioral intervention for self-management of mental health (anxiety, depression, and low mood) Duration: 4 weeks	AI chatbot (Elomia; NLP^ak^+ML based) Platform: not identified	Young adults with depression, anxiety, and low mood N=412 (202 female and 210 male) Participants aged ≥18 years (19-23 years)	Pretest-posttest observational study—effectiveness evaluation using psychological quantitative research methods through a controlled experiment in 3 stages: formation of experimental and control samples, baseline testing, and final testing Location: Ukraine	Significant reduction in the high tendency toward depression (up to 28%) Significant reduction in the high tendency toward anxiety (up to 31%) Significant reduction in the high tendency toward negative effects (up to 15%) through the regular use of Elomia	Relatively small sample size and focus on specific student age group may limit generalizability of study findings to other populations Short 4-week study duration may be insufficient to assess Elomia’s long-term effectiveness No follow-up assessment may restrict evaluation of Elomia's sustainability Absence of qualitative analysis of user feedback may limit UX insights Lack of comparison with other web-based psychological services or traditional face-to-face therapy may produce a less comprehensive evaluation of the chatbot’s effectiveness
Anan et al [98], 2021	Lifestyle intervention to support adherence to exercises for self-management and improvement in musculoskeletal symptoms Duration: 12 weeks	AI chatbot Platform: mobile devices or smartphones via a messaging app (LINE)	Employees with musculoskeletal symptoms (eg, neck and shoulder stiffness and low back pain) and smartphone users N=121 (intervention group: n=61; control group: n=60) Participants’ age not specified	Pretest-posttest (quantitative) observational study—2-arm RCT following CONSORT-EHEALTH^al^ guidelines Location: Japan	Primary outcomes: Statistically significant improvement (*P*<.001) in the average pain level (on a scale of 1 [highest] to 5 [lowest]) of the neck and shoulder stiffness or pain or low back pain in the intervention group (average pain level=3.0, SD 1.1), compared to the control group (average pain level=4.0, SD 0.8) Proportion of participants with severe symptoms significantly decreased from 77% (37/48) to 33% (16/48) in the intervention group, whereas the decrease in the control group was from 76% (33/46) to 67% (31/46) Significant improvements in the severity of the neck and shoulder pain and stiffness and low back pain in the intervention group compared to the control group (OR^am^ 6.36, 95% CI 2.57-15.73; *P*<.001) Subjective assessment: 75% in the intervention group showed symptom improvement, whereas only 3% in the control group showed improvement (*P*<.001) Secondary outcomes: The AI-based health intervention was effective in improving the EQ-5D-5L score and the RMDQ^an^ score in the intervention group	Limited generalizability due to single-company study setting No information collected on causes, diagnosis, and treatment status of neck and shoulder pain and low back pain Pain improvement observed in the intervention group may be influenced by treatment changes and occupational factors, not only by the intervention. Lack of long-term follow-up to assess intervention sustainability
Zisis et al [99], 2021	To provide patient education and support self-management of acute decompensated HFao Duration: 12 weeks	AI-based avatar (HF-Coach; NLP+ML based) Platform: mobile devices or smartphones and tablets	Patients with chronic acute decompensated HF N=200 Participants’ age: mean 55 years	RCT—quantitative study using patient medical records and oral interviews Location: Australia	No significant differences between the intervention and control groups in mood (GAD-7^s^ and PHQ-9), cognition (MoCA^ap^), HF knowledge (DHFKS^aq^), or HRQoL Enrolled participants had better self-care behavior Barriers to implementation identified: lack of interest, inadequate technological and computer literacy, language limitations, anxiety caused by app questions, data retrieval issues, and disabilities (eg, visual, hearing, and cognitive impairments)	Small enrollment numbers Low patient engagement High dropout from the HF app Findings based on experiences from a single trial
Stara et al [73], 2021	To support self-management of dementia in the home environment Duration: 4 weeks	3D-animated human-like AI-based avatar (Anne) Platform: mobile device or smartphones Microsoft Surface Pro tablet	Older adults with dementia and their caregivers N=20 (30% male and 70% female) Participants aged ≥65 years (mean 75.5, SD 4.2 years)	Usability and acceptability evaluation study—mixed methods approach Location: Italy	Usability (SUS) score: 67.1 among older adults and 71.4 among caregivers, indicating acceptable usability No significant change in quality of life in older adults with cognitive impairment (QoL-AD^ar^) before or after the study (mean change 0.4, SD 4.6) 42% (8/20) of older adults’ perceived closeness with Anne as feeling some overlaps, while 26% (6/20) reported feeling no overlap, and 11% (2/20) felt strong, equal or little overlap	Small sample size and Italian national context and culture may limit result generalization Short 4-week study duration may be insufficient to significantly evaluate acceptability and usability Technical discomfort of participants with automatic speech recognition
Nguyen et al [74], 2021	Self-management of diabetes through patient education on diabetes care, blood glucose monitoring, and managing diabetes complications Duration: 8 weeks (52 days)	Multilingual (text+voice enabled) AI bot (AMANDA; ML based using deep learning) Platform: mobile devices or smartphones	Patients with diabetes who need self-care N=20 (nurses and clinicians) Participants’ age not identified	Usability and quality evaluation of AMANDA—a quantitative survey questionnaire filled out by 20 judges after listening to the real and TTS^as^-generated audio samples Location: Singapore	Naturalness: 4.07 Accent uniqueness: 3.98 Information clarity: 3.88 SUS score: 80.625 (above the average score of 68); 70% of the participants gave a score of ≥80 (indicating high usability of the interface)	Small sample size and language restriction (availability in English only) may limit result generalization Laboratory setting evaluation may not reflect real-world use Impact of the CA on users’ health outcomes (eg, blood glucose levels and medication adherence) and their emotional well-being or quality of life was not considered in the evaluation
Apergi et al [100], 2021	Self-care and communication improvement between patients and health care providers Duration: 90 days	Commercial voicebot (Alexa) and a voice-enabled, animated avatar Platform: Alexa+: smart speaker device (Amazon) Avatar: tablets	Patients with HF N=55 (Alexa+: n=28; avatar: n=27) Participants’ age not identified	Quantitative pilot comparison study—demographic and technology survey and daily questionnaires Location: not identified	Positive correlation between patients’ age and the technology use (coefficient=1.19; *P*=.004) No statistically significant difference in engagement levels between the avatar and Alexa+ user groups Decrease in use over time for both technologies, with a sharper decrease observed for Alexa+ participants Black patients with similar characteristics used the technology 21 fewer times on average compared to non-Black patients (coefficient=–15.96; *P*=.08) Results indicate that technology design may need to be better tailored for Black patients with HF	Small sample size and focus on specific patient populations may limit generalizability of the findings Potential bias due to exclusion of possible important variables or unaccounted confounding factors in the model Lack of normalization of data for control variables Limited exploration of reasons for differences in technology engagement among Black patients
Kataoka et al [81], 2021	Personalized patient education for self-management of lung cancer symptoms Duration: 1 month	AI chatbot (rule-based AI using Google Cloud’s Dialogflow and predetermined keywords) Platform: mobile devices or smartphones and web browser (via LINE app)	Patients with lung cancer and their caregivers N=12 (11 patients and 1 caregiver) Participants’ age not identified	Feasibility and usability study—sequential mixed methods approach through a web-based qualitative survey questionnaire and quantitative alpha and beta testing of chatbot with stakeholders Location: Japan	The chatbot was found to be feasible but inadequately used Mean user satisfaction score was 2.7/5, indicating low user satisfaction Chatbot was able to deliver appropriate responses to most FAQs^at^ and also identify areas where additional responses were required 82 categories of FAQs and formulated responses to these FAQs were identified, which were used to develop the chatbot Potential of chatbot to improve patient knowledge of symptom management was demonstrated	Small sample size and single-hospital setting may limit applicability of study findings 8 questions did not match well with responses in phase 5, leading to patient dissatisfaction Questions for nonexistent categories remained unmatched, indicating the need to add educational categories and responses through further discussion
Rathnayaka et al [101], 2022	Behavioral intervention for remote management of mental health issues Duration: 8 weeks	AI chatbot (Bunji; rule based+ML) Platform: mobile devices or smartphones	Individuals with mental health issues N=318 Participants’ age not identified	Mixed methods pilot study—participatory evaluation through quasi-experimental design and qualitative feedback Location: Australia	Participatory evaluation (through 3 experimental pilot studies) Study 1: positive impact in improving users’ mood using Bunji's emotional support features Study 2: positive impact of personalized conversation on improving the effectiveness of Bunji in providing mental health support to users Study 3: positive feedback on the features of the chatbot and its effectiveness in providing remote mental health support to users	Limited sample size may impact result generalizability Short study duration (8 weeks) may be inadequate to capture long-term effects or user behaviors Reliance on users’ self-reported data such as mood scores and survey responses could introduce bias Chatbot’s performance could be affected by technical issues such as connectivity or device compatibility Lack of a control group may limit the ability to attribute changes to the chatbot’s intervention
Kannampallil et al [102], 2022	To provide personalized and accessible mental health care with realistic and cognitively plausible verbal interaction Duration: not identified	AI voice-based coach and voicebot (Lumen) Platform: Amazon Alexa	Individuals with mild to moderate depression and anxiety seeking behavioral therapy N=26 Participants’ age: mean 43.9 years	Mixed methods observational study: Efficacy evaluation—RCT Acceptability and usability evaluation with WAI-TECH^au^ survey Location: not identified	Task load (mental and physical effort required to complete a task): medium workload (higher temporal workload in session 2 [mean Task subscale 5.3, SD 0.9] than in session 1 [mean Task subscale 5.2, SD 0.9]) Work alliance (collaborative relationship between therapist and client): moderately high, indicating well-aligned sessions with participants’ needs, potential goals (session 1-mean Goal subscale 5.0, SD 0.9; session 2- mean Goal subscale 5.1, SD 0.9), and mutual liking between participants and voicebot (session 1-mean Bondscale 4.9, SD 1.0; session 2-mean Bondscale 4.7, SD 1.0) Participants highlighted lack of personalization, depth, and emotional engagement in the conversations Overall UX: positive evaluation (values of >0.8) for pragmatic, hedonic, and overall qualities related to UX with Lumen for both sessions	Small sample size of users in a relatively controlled environment, potentially limiting applicability of findings Evaluation limited to 2 sessions, not representing the full 8-session PST^av^ program Potential influence of research coordinator and note taker on participant responses and Lumen use Inability to assess impact of various measures (task load and work alliance) over time due to study constraints Technological limitations of current AI-based voice technology include challenges in understanding natural language and interpreting emotional cues in voice interactions
Rahmanti et al [78], 2022	Lifestyle intervention for weight management through empathetic persuasive conversation flows Duration: 7 days	AI (empathetic) chatbot (SlimMe; rule based+ML using Dialogflow) Platform: mobile devices or smartphones	Individuals intending to lose or maintain weight N=10 (100% female) Participants aged 24-34 years	Mixed methods usability study through simulation trial—UX questionnaire after the trial Location: Taiwan	Positive chatbot UX: ease of use; usefulness; and fun attributes such as use of emoticons, stickers, and GIF^aw^ images Negative chatbot UX: slow response time and irrelevant responses	Small sample size and language restriction (only English) may limit result generalization Nutrition assessment methods rely on user self-reports, which may introduce bias Self-reported anthropometric measurements may be less accurate compared to direct measurements
Alturaiki et al [103], 2022	To improve self-management of β thalassemia and communication between patients and health care providers Duration: not identified	AI chatbot (rule based) Platform: web browser on computers or mobile devices	Patients with β thalassemia N=34 Participants’ age not identified	Prototype implementation and usability evaluation study through qualitative posttest survey Location: not identified	Usability and perceived utility assessment: Most participants (72%) found the chatbot easy to use Above 90% of participants considered the chatbot beneficial Most participants agreed that the chatbot made managing β thalassemia easier and more efficient Chatbot has the potential to save patients time and money that they usually spend on hospital visits	Small sample size, which may limit generalizability of findings Chatbot was tested in a live chat scenario, possibly not reflecting real-world use accurately Limited data availability, with findings not publicly accessible due to privacy or ethical constraints No specification of statistical analysis or validation methods for assessing the chatbot’s effectiveness Long-term usability and effectiveness of the chatbot in managing β thalassemia was unexplored
Zahedi et al [104], 2022	To support constant ubiquitous medical care for GI^ax^ disease management through an avatar-based telepresence system Duration: not identified	Virtual human avatars (AI nurse avatar and digital patient avatars) in a virtual hospital system (Wepital) Platform: computer (laptops and desktop computers) via the Second Life website	Patients with GI problems N=61 Participants’ age not identified	Hypothesis experiment and usability study with real patients using the Wepital prototype—mixed methods approach Location: not identified	Significant positive impact of telepresence affordance on patient satisfaction with the Wepital system, with a path coefficient of 0.41 (*P*<.001) Significant positive impact of convenience affordance on patient satisfaction with the Wepital system, with a path coefficient of 0.33 (*P*<.001) Significant positive impact of trust affordance on patient satisfaction with the Wepital system, with a path coefficient of 0.43 (*P*<.001) Significant positive impact of using real avatars with wearable sensors on patient satisfaction with the Wepital system, with a coefficient of 0.11 (*P*<.01) Significant positive impact of patients’ predispositions, such as flexibility regarding method of care and understanding information, on their perceptions of affordances in the Wepital system, with coefficients of 0.70, 0.63, and 0.29 (*P*<.001 in all cases), respectively No significant influence on trust due to lack of privacy concern	Small sample size due to limited patient participation because of sensitive context and strict recruitment rules Researchers’ restricted access to patients’ medical records prevented the integration of real avatars with comprehensive medical information Study focused on patients with GI in a single context, limiting generalizability of findings to other medical fields Patients staying in the Wepital for persistent care were not involved in the study, limiting understanding of patient satisfaction
Eagle et al [37], 2022	Evaluation of CA’s mental health–related advice for improved self-management of depression and anxiety Duration: not identified	6 commercial CAs—chatbots (Wysa and Replika) and voicebots (Google Assistant, Alexa, Cortana, and Siri) Platform: Chatbots: mobile devices or smartphones Voicebots: smart speaker devices	People experienced using CAs for mental health advice and management N=141 Participants’ age not identified	Mixed methods observational study—response quality evaluation using user-bot conversational data, PHQ-9 and GAD-7 survey questionnaires, and interviews with clinicians Location: United States	Generally low quality of advice and recommendations provided by CAs for mental health questions Wide variability in the quality of responses across different CAs—no significant advantage of voice agents over chatbots Text-based chatbots slightly outperformed voice-based agents with improved responses regarding better treatment advice due to the chatbots’ superior dialogue capabilities and empathy support, whereas voice assistants were prone to speech recognition errors and were more dependent on simple web searches in generating advice CAs struggled to handle unexpected inputs Anxiety-related responses were slightly better than the responses for depression and crisis situations Extended dialogue context access improved the CAs’ quality of responses Design implications included providing clarification of subdialogues and access to extended dialogue context	Limited sample size may restrict the generalizability of findings to a wider population Assessment of CAs’ responses limited to mental health questions on anxiety and depression Standardized survey-based conversational probes may not have captured the full range of user queries and responses in real-world settings Quality of responses assessed through subjective participant ratings, introducing potential biases No investigation on the impact of user characteristics (eg, age, gender, or previous mental health service experience) on response quality Long-term effects of CAs’ advice on user outcomes such as mental health improvement or treatment adherence were not addressed Ethical implications of using CAs for mental health advice, including privacy concerns and potential harm from inaccurate information, were unexplored CAs’ responses were not compared with those provided by human experts, which could have provided insights into effectiveness
Maharjan et al [83], 2022	Self-report of depression or bipolar disorder Duration: 4 weeks	Voice assistant and voicebot (Sophia; rule-based AI using Dialogflow) Platform: Google Nest Mini smart speakers	Adults with depression or bipolar disorder N=20 Participants aged ≥18 years (18-34 years)	Mixed methods in-the-wild study—acceptability and usability evaluation using user-agent engagement data, fortnightly completed WHO-5 health questionnaire, and semistructured interviews Location: not identified	Global average engagement rate=75%, indicating a high engagement level with the voicebot Diverse UX with varying personified perceptions, social context, privacy and security concerns, and conversational features Perceived helpfulness of self-reporting practice in daily reflection and through organization, and the process was considered meditative by some participants The voicebot was perceived as usable and acceptable for self-reporting mental health well-being in a naturalistic setting	Study conducted during the pandemic, potentially impacting participants’ mental, physical, and social conditions, affecting result generalizability Participants’ interaction with the CA may be influenced by lockdowns, social distancing, and travel restrictions, affecting their typical context and experiences Interview method (web-based or in person) could influence how participants conveyed their experiences, potentially influencing study outcomes Privacy and data security concerns among participants, leading to self-censorship during self-reporting Participants’ engagement and experiences with the CA may be affected by their varying technology familiarity levels
Meheli et al [105], 2022	Cognitive behavioral intervention for self-management of chronic pain and mental health Duration: not identified	Commercial CA—AI mental health chatbot (Wysa) Platform: mobile devices or smartphones	Individuals with self-reported chronic pain seeking digital mental health support N=2194 (real-world data) Participants’ age not identified	Mixed methods retrospective observational study—chatbot effectiveness evaluation through user-chatbot engagement data and pretest-posttest assessment questionnaires (PHQ-9 and GAD-7) Location: not identified	Identified themes related to health concerns, socioeconomic concerns, and pain management concerns among users with chronic pain Users with chronic pain showed significantly higher app engagement (*P*<.001) compared to users without chronic pain, with a large effect size (Vargha and Delane A=0.76-0.80) Significant reduction in anxiety and depression symptoms among users with chronic pain, as shown by the pretest-posttest assessments using PHQ-9 and GAD-7 scales (*P*<.001), with a medium effect size (Cohen *d*=0.60-0.61) Chatbot was found to be useful in managing challenges, including anxiety, sleep, low energy, motivation, loss, and pain Conversational data revealed perceived needs and experiences of individuals with chronic pain, including the need for personalized and flexible support	Small samples for the third objective may further constrain generalizability of findings, underscoring the need for careful interpretation as preliminary outcomes Nonrandomized sampling design may limit generalizability of findings Data extraction keywords based on guidelines, literature, and clinical experience may have missed relevant pain terms Repeated measurements for efficacy without a control group might risk increasing regression to the mean Users were not mandated to complete assessments, limiting efficacy study to a small subset
Henson et al [106], 2022	To provide appropriate patient education for self-management of GERD^ay^ Duration: not identified	Commercial CA—AI chatbot (ChatGPT) Platform: multiple devices—mobile devices or smartphones, laptops, and desktop computers (web browser and app)	Patients with GERD N=11 (8 patients and 3 gastroenterologists) Participants’ age not identified	Feasibility study (quantitative)—potential utility evaluation of ChatGPT in GERD using 23 GERD management prompts Location: not identified	63/69 (91.3%) appropriate responses to GERD management queries delivered by ChatGPT Frequent inconsistency in responses to the same prompt, with 16/23 (70%) prompts resulting in variable appropriateness Highest number of appropriate responses (39.4%) was to treatment-related prompts, whereas the highest number of inappropriate responses (14.3%) was to diagnosis and management–related prompts Most responses (78.3%) contained some specific guidance, particularly for diagnosis-related prompts (93.3%) Patients with different educational backgrounds considered the responses understandable and more useful than a search engine ChatGPT failed to recommend consideration of Roux-en-Y gastric bypass for ongoing GERD symptoms with pathological acid exposure in obesity setting Current limitations prevent the integration of ChatGPT into routine clinical practice at present	Small sample size may limit the generalizability of findings ChatGPT was not specifically trained on medical literature, and its ability to address repeated requests for clarification was not evaluated Inappropriate responses with inconsistencies to the same prompt were observed, which may affect results Limited specific guidance and content errors PPI^az^ risks were stated as fact, which lacked balanced consideration of benefits and context ChatGPT often provided overly long responses, diluting the clinical impact of responses in many cases
Babington‐Ashaye et al [75], 2023	To improve patient knowledge and support symptom monitoring of hemophilia Duration: not identified	AI chatbot (Saytù Hemophilie; NLU^ba^ and ML based) Platform: mobile devices or smartphones (via messaging apps such as WhatsApp, Signal, and Telegram)	People with hemophilia and their families in Senegal N=30 (20 people with hemophilia and 10 family members) Participants’ age not identified	Usability study—mixed methods approach with SUS survey Location: Senegal (West Africa)	Average usability (SUS) score: 81.7 (indicates high usability of the system) 75.4% of participants expressed an overall high level of satisfaction with the French version of the chatbot Most participants perceived the proposed AI-based chatbot as a potential solution to manage the symptoms at home while awaiting a physician’s consultation	Small sample size restricted the ability to conduct additional statistical tests, limiting result generalizability Potential sample bias as 40.6% of participants recruited were students While the SUS score was used, additional evaluation measures could be used to assess users’ overall opinion of the chatbot
Alhmiedat and Alotaibi [79], 2023	Symptom monitoring and patient education for T1DM management Duration: 5 days	Humanoid robot (SARA) Platform: physical presence	Children with T1DM N=5 (children) Participants aged 5-9 years	Pilot study—acceptability evaluation of SARA using quantitative questionnaires, observations, and experiments Location: Saudi Arabia	All participants succeeded in all the following measures with different outcomes: Total interaction time with SARA Productivity of education task Productivity of the short test task Productivity of listening to stories Moderate worthiness level: 88.2%, indicating a high relative acceptability level User feedback: positive feedback from the patients, their parents, and clinical staff	Very small sample size of the pilot study, limiting generalizability of results SARA framework is still under development, requiring further experiments before finalizing the robotic platform
Calvo et al [84], 2023	To estimate the risk of an asthma exacerbation and provide recommendations for improved self-management of asthma Duration: 28 days	Text-based chatbot (rule-based AI) Platform: mobile devices or smartphones (WhatsApp based)	Adults with asthma N=300 (40 for alpha testing and 260 for beta testing) Participants aged ≥18 years	Feasibility and usability study with pretest-posttest quantitative questionnaires Location: United Kingdom	Chatbot was found to be acceptable, usable, and satisfactory to participants as it was helpful in improving their asthma control Task completion rate: 80% of participants completed the risk assessment process, whereas 20% dropped out Net Promoter Score for the chatbot: 8.5 (indicating high likelihood of recommending it to others with asthma) Participants reported that they were satisfied with the chatbot and that the risk measurement by the chatbot was useful Participants suggested improving the chatbot by incorporating more personalized feedback and additional interactive features Chatbot seemed to be effective in promoting motivational quality, engagement, and UX in relation to basic psychological needs High level of alignment between expected and calculated risk, and the calculated risk was perceived as accurate by the user	Limited sample size No intention to find gender or age differences within the current sampling No intention to measure significant asthma control improvements in 28 days Potential for incorrect responses from individuals who incorrectly answered the screening questionnaire Lack of validation for measurable health outcomes
Epalte et al [107], 2023	Personalized exercise program with patient education and counseling support for self-management of poststroke recovery Duration: 1 month	AI chatbot (Vigo; rule based+ML) Platform: smart devices (eg, Apple iPad tablet)	Patients with stroke and their families N=12 Participants’ age not identified	Usability study—qualitative semistructured interviews after the intervention Location: not identified	Participants considered the chatbot transparent, understandable, and handy Overall design of Vigo was rated as good Participants were mostly unsatisfied with the difficulty level and diversity of exercises 5 themes related to the app’s effectiveness were identified: flexibility, information, exercises, emotional support, and assessment Chatbot could have a potential positive impact on poststroke outcomes, particularly in emotional status, social interaction, improved mood, and motivation	Strict inclusion criteria and chatbot’s complexity limited recruitment of patients with greater physical limitations, potentially skewing insights Patients with severe functional limitations, aphasia, cognitive deficits, and comorbidities may struggle to use the app independently, reducing the potential user base Chatbot’s availability only in Latvian may restrict use for Russian-speaking patients Difficulty in adapting the program for patients due to their different health statuses, personal factors, and preferences
LeRouge et al [108], 2023	Personalized lifestyle intervention for self-management of obesity Duration: not identified	AI-based computer-animated cartoonlike 2D avatars Platform: mobile devices or smartphones and desktop computers	Adolescents with obesity or overweight N=70 (for phase 2) Participants’ age not identified	Qualitative participatory research study in 2 phases: Phase 1: focus groups with teenagers, provider interviews, and parent interviews Phase 2: midrange prototype assessment by teenagers and providers Location: United States	Effectiveness: avatars and virtual agents can be effective in engaging adolescents in weight management programs Avatars and virtual agents can provide personalized support and motivation to adolescents Users would be more motivated to use the virtual agents if they were more fun, creative, human-like, interactive, communicative, and indicative of progress or success Design characteristics: Personalize avatars matching user needs and preferences Ability to stimulate 2-way interaction (preferably fun, creative, and more human-like) Support multiple communication modes (eg, text, voice, and video) Provide feedback on user progress and behavior Challenges: Implementation of avatars requires technical expertise and resources User acceptance barriers due to trust issues and privacy concerns Integration requirements of avatars with existing health care systems and workflows Development of avatars and virtual agents can be expensive	Limited data sources may limit universal applicability of findings Study scope was limited to a specific audience using qualitative methods, which may limit generalizability Potential coding bias in the data analysis process despite minimization efforts
Park et al [109], 2023	Improvement of web-based mental health counseling efficacy through effective conversation between a user and a chatbot agent Duration: not identified	2 AI chatbots (human-like vs machinelike; NLP using Dialogflow) Platform: not identified	Adults seeking web-based mental health support N=385 Participants’ age not identified	Quasi-experimental design—quantitative UX evaluation of a human-like vs machinelike chatbot for web-based mental health counseling Location: United States	Human-like chatbot apparently yielded higher user intention to comply with health recommendations (mean 5.69, SD 0.61) than the machinelike one (mean 5.34, SD 0.93; t383=4.41; *P*<.001) Human-like chatbot resulted in higher compliance with health recommendations mediated by psychological distance Positive correlation between human representation and perceived trust and between perceived trust and compliance with health recommendations Human-like chatbot led to psychological closeness and increased trust, thereby causing higher intention to comply with health recommendations	Convenience sampling could limit generalizability Study focused solely on chatbot’s physical representation, excluding other important cues such as conversational and identity cues User characteristics such as involvement and self-efficacy, which could moderate the impact of human-like cues, were unexplored Effect of demographic variables (eg, age, gender, and ethnicity) was not investigated for both users and chatbot agents Using a self-administered web-based questionnaire might introduce limitations such as social desirability bias and response bias while lacking control over participants’ environments Long-term effects of chatbot counseling on mental health outcomes were not investigated
Boggiss et al [110], 2023	To deliver self-compassion coping lessons to support T1DM self-management and mental health Duration: not identified	AI self-compassion chatbot with customizable avatar (COMPASS) Platform: mobile devices or smartphones	Adolescents with T1DM N=30 (19 adolescents with T1DM and 11 diabetes health care professionals) Participants aged 12-16 years	Qualitative study—focus groups and interviews Location: New Zealand	COMPASS chatbot was found to be acceptable to adolescents with T1DM and diabetes health care professionals The chatbot apparently had the potential to support the T1DM self-management and mental health of adolescents during the COVID-19 pandemic Participants emphasized the importance of personalization of both content and features of the chatbot (eg, games, apps, background color schemes, and hobbies) and cultural appropriateness to support users with different ethnic backgrounds	Small sample size may not be representative of the breadth of challenges faced by adolescents with T1DM or additional barriers to standard care access Potential bias in user feedback due to existing relationships between the facilitator and participants Feedback was limited as participants only viewed screen recordings of the chatbot, potentially reducing its richness Primary assessment limited to acceptability, perceived clinical utility, and usability of chatbot features, potentially overlooking the intervention’s overall impact User feedback may be influenced by inequities in access to technology such as smartphones and continuous glucose monitors

^a^CA: conversational agent.

^b^AI: artificial intelligence.

^c^RCT: randomized controlled trial.

^d^AF: atrial fibrillation.

^e^AFEQT: AF Effect on Quality of Life questionnaire.

^f^T2DM: type 2 diabetes mellitus.

^g^ML: machine learning.

^h^mHealth: mobile health.

^i^AADE: American Association of Diabetes Educators.

^j^WHO-5: 5-item World Health Organization Well-Being Index.

^k^K10: Kessler Psychological Distress Scale.

^l^BADS: Behavioral Activation for Depression Scale.

^m^BADS-AC: Behavioral Activation for Depression Scale-Activation.

^n^BADS-AR: Behavioral Activation for Depression Scale-Avoidance/Rumination.

^o^BADS-WS: Behavioral Activation for Depression Scale-Work/School Impairment.

^p^BADS-SI: Behavioral Activation for Depression Scale-Social Impairment.

^q^VDMS: virtual diabetes management system.

^r^PHQ-9: 9-item Patient Health Questionnaire.

^s^GAD-7: 7-item Generalized Anxiety Disorder Scale.

^t^T1DM: type 1 diabetes mellitus.

^u^PAL: personal assistant for a healthy lifestyle.

^v^SUS: System Usability Scale.

^w^BCT: behavior change theory.

^x^HRQoL: health-related quality of life.

^y^AQoL-8D: Assessment of quality of life-8 dimensions.

^z^HbA_1c_: glycated hemoglobin.

^aa^HADS: Hospital Anxiety and Depression Scale.

^ab^GP: general practitioner.

^ac^SCD: sickle cell disease.

^ad^EVA: Education virtual assistant.

^ae^FBG: fasting blood glucose.

^af^PPBG: postprandial blood glucose.

^ag^VH: virtual human.

^ah^UX: user experience.

^ai^UEQ: User Experience Questionnaire.

^aj^TAM: technology acceptance model.

^ak^NLP: natural language processing.

^al^CONSORT-EHEALTH: Consolidated Standards of Reporting Trials of Electronic and Mobile Health Applications and Online Telehealth.

^am^OR: odds ratio.

^an^RMDQ: Roland-Morris Disability Questionnaire.

^ao^HF: heart failure.

^ap^MoCA: Montreal Cognitive Assessment.

^aq^DHFKS: Dutch Heart Failure Knowledge Scale.

^ar^QoL-AD: Quality of Life in Alzheimer Disease scale.

^as^TTS: Text-to-Speech.

^at^FAQ: frequently asked question.

^au^WAI-TECH: Working Alliance Inventory–Technology Version.

^av^PST: problem-solving treatment.

^aw^GIF: graphics interchange format.

^ax^GI: gastrointestinal.

^ay^GERD: gastrointestinal reflux disease.

^az^PPI: proton-pump inhibitor.

^ba^NLU: Natural Language Understanding.

A few studies (6/43, 14%) focused on evaluating the effectiveness of the CAs used for the self-management of certain chronic illnesses, such as atrial fibrillation [10], type 2 diabetes mellitus [24,82], and mental health conditions such as depression and anxiety [90,96,97]. All these studies found improvement in various parameters in management, such as significant reduction in glycated hemoglobin level (*P*<.001), as well as BMI, and weight (*P*<.001) in patients with type 2 diabetes mellitus [82]; notable improvement in quality of life measured using the Atrial Fibrillation Effect on Quality of Life questionnaire score (*P*<.05) or health-related quality of life utility score (*P*=.007) in patients with atrial fibrillation [10,24]; and substantial reduction in depression (9-item Patient Health Questionnaire score) and anxiety (7-item Generalized Anxiety Disorder Scale score) symptoms [90,97,105].

### Types of AI-Based CAs in the Included Studies

Of the 47 AI-based CAs representing digital health assistants identified from the included studies, 9 (19%) were existing commercial CAs available as marketed products, which included voice assistants—Google Assistant, Amazon Alexa, Microsoft Cortana, and Apple’s Siri, as well as social chatbots—ChatGPT and Replika (a voice-enabled, avatar-based chatbot), and health care chatbots—Vik, Wysa, and Tess. The remaining 38 CAs were proposed designs or prototypes, where 19 (50%) of them were chatbots, 2 (5%) were voice assistants or voicebots [58,102], 13 (34%) were human-like digital avatars, 1 (3%) was a digital avatar version of a humanoid social robot [85], 1 (3%) was a text-based voice-enabled bot [74], and 2 (5%) were avatar-based chatbots [91,110]. In addition, 5% (2/38) were CAs as humanoid social robots [79,85], which were included as they particularly emphasized managing NCDs such as diabetes (although the *robots* topic was beyond the scope of this study).

### Intelligence Level of the AI-Based CAs

Of the 38 CA prototypes presented, 27 (71%) were found to have indicated their AI algorithm implementation methods to generate appropriate health-related responses. A total of 30% (8/27) of them were implemented using rule-based algorithms [72,81,83,84,86-88,103], 7% (2/27) of the CAs were developed using network-based scripted algorithms [10,80], and 4% (1/27) of the CAs were implemented using prescripted algorithms based on behavior change theory, whereas 33% (9/27) of the CAs were implemented using ML approaches [58,74,75,89,93-95,97,99] and 15% (4/27) of the CAs were developed using a combination of rule-based and ML techniques [78,85,101,107]. However, one of the CAs identified from a study was a Wizard-of-Oz human-like AI avatar with prescripted text and human-controlled responses to evaluate the potential effectiveness of visually embodied avatars in the assessment of anxiety and depression through users’ behavioral expressiveness [96].

### Types of Health Interventions and Study Duration

Most included studies (24/43, 56%) focused on behavioral or lifestyle interventions and patient education for improved self-management of physical NCDs such as *CVDs* [10,80,99,100], *diabetes* [24,72,74,79,82,85,89,93] or *prediabetes* [76], *cancer* [81,92], *gastrointestinal diseases* [104,106], and obesity [76,86,108].

A reasonable number of studies (13/43, 30%) focused on cognitive behavioral interventions related to the self-management of chronic cognitive impairments (eg, dementia and poststroke) [73,107], including chronic mental health conditions such as anxiety, depression, psychological distress, and bipolar disorders [83,87,88,90,96,97,101,102].

The duration of these studies was reasonably divergent, ranging from 5 days to 1 year. However, a substantial number of studies (14/43, 33%) could be considered long term, such as 8 weeks to 2 months [85,88,90,99,101], 12 weeks to 90 days [86,98,100], 16 weeks to 6 months [76,82,87,93], 9 months [94], and 12 months or 1 year [24,92]. A total of 26% (11/43) of the studies conducted trials for approximately ≤1 month [10,73,81,83,84,88,97,107] (ie, within 5-10 days) [72,78,79]. A total of 42% (18/43) of the studies did not mention the study duration.

### Study Population and Location

Almost half (20/43, 47%) of the included studies did not specify the target population other than patients with a particular chronic disease that the health intervention was meant for. The studies that specified the target population or their age group (24/43, 56%) were relatively heterogeneous, with older adults [10,58,72,73,91,95] and middle-aged adults [24,99] being the most frequently targeted user group (8/43, 19%). However, some studies (6/43, 14%) also focused on younger adults [97], adolescents [76,108,110], and children [79,85].

The locations of the studies conducted were largely diverse, but most (27/43, 63%) were conducted in higher-income countries. A total of 9 studies were completed in Europe, where 2 (22%) of them were conducted in the United Kingdom [84,91], 2 (22%) studies were conducted in Italy and the Netherlands [73,85], 1 (11%) was conducted in Portugal [72], 1 (11%) was conducted in France [77], 1 (11%) was conducted in Greece [93], 1 (11%) was conducted in Spain [94], and 1 (11%) was conducted in Ukraine [97]. A total of 16% (7/43) of the studies were conducted in the United States [10,37,76,86,87,108,109]. In total, 4 of the studies were carried out in the Australasia region—3 (75%) in Australia [24,99,101] and 1 (25%) in New Zealand [110]. A total of 7 studies were conducted in Asia, where 4 (57%) of them were conducted in Japan [81,88,96,98], 1 (14%) was conducted in Singapore [74], 1 (14%) was conducted in Taiwan [78], and 1 (14%) was conducted in India [82]. A total of 5% (2/43) of the studies were conducted in Saudi Arabia [79] and Senegal in West Africa [75]. The remaining 33% (14/43) of the studies did not specify the study location.

### Data Collection Methods of the Included Studies

The included studies mainly used quantitative (17/43, 40%) or mixed methods (17/43, 40%) approaches. A substantial proportion of these studies (11/43, 26%) were pretest-posttest intervention studies to observe the differences before and after the intervention within the same group of participants (single armed) or between intervention and control groups (2 armed). The study designs of the quantitative and mixed methods studies included observational experiments using 2-armed randomized controlled trials (7/34, 21%) [10,24,86,87,98,99,102], a nonrandomized prospective study (1/34, 3%) [88], retrospective studies (2/34, 6%) [82,105], quasi-experimental studies (4/34, 12%) [10,90,101,109], a single-armed pilot trial [94], and comparative evaluation studies (3/34, 9%) [89,96,100].

The qualitative studies (9/43, 21%) were mostly usability and acceptability assessments of prototypes through qualitative posttest surveys [103], postintervention interviews [107], focus groups [110], a participatory design approach with focus groups and interviews [108], and workshops [91]. A total of 22% (2/9) of the qualitative studies compared the proposed prototypes against similar existing applications [58,89].

## Discussion

### Principal Findings

The discussion of this scoping review synthesizes the findings in light of the overarching themes emergent from each research question, elucidating the interplay between the current state, challenges, benefits, and the targeted users of AI-based CAs within the scope of the examined literature.

#### Current State of AI-Powered CAs

Regarding the current state of research on AI-powered CAs as human-like health carers for managing NCDs, empirical studies have revealed the limited ability of existing commercial CAs to provide appropriate lifestyle advice and health recommendations [37,111]. Perhaps this is due to the lack of specified data training for these commercial tools in the medical context, which is currently restricting their integration in clinical and remote care [106].

Functionality-wise, commercial chatbots (eg, Wysa and Replika) tend to slightly outperform voice agents (eg, Google Assistant, Siri, Alexa, and Cortana) with improved response quality due to the former’s better dialogue capabilities and empathy support. At the same time, the latter are more susceptible to speech recognition errors, including greater dependency on simple web searches to generate advice [37]. Nonetheless, voice-enabled CAs offer hands-free communication, making them preferable over text-based chatbots for older adult users, as suggested by a recent scoping review conducted by Even et al [70] focusing on the benefits and challenges of CAs in the older adult population (Table 1). In addition, another study revealed that older adults preferred voice-based communications over SMS text messages or free-text entry as voice is regarded as a powerful mode for promoting motivation [112]. In addition, voice agents may also be more accessible compared to digital avatars due to their lower bandwidth requirements, making them suitable for locations with lower bandwidth connectivity or devices with limited computing power [48]. Conversely, digital avatars are generally more likely to be favored by users over other types of CAs for their widely accepted face-to-face conversation format [10] as they can support both text and audio outputs [47,113] and, therefore, can serve as a valuable alternative complement for remote care assistance [114]. This suggests that digital avatars remain a popular choice for their versatility and interactive capabilities, driving the rapid progression toward the anthropomorphism era as they represent realistic human-like traits in behaviors and appearances for enhanced interaction with the users [115].

Moreover, anthropomorphic CAs are more preferred over nonanthropomorphic CAs, as shown by Park et al [109], indicating that the human-like representations of a chatbot yielded a higher likelihood of intervention compliance than the machinelike chatbot because the former increased closeness and trust. Similarly, another study demonstrated that an augmented human-like embodiment of a digital avatar resulted in greater social closeness among the participants [116]. Correspondingly, strong social presence and emotional closeness are regarded as key facilitators of favorable attitudinal and behavioral outcomes among users [117]. Hence, anthropomorphism positively correlates to users’ perceived trust and acceptance of a virtual agent [12,117]. Indeed, digital human faces as intelligent CAs are considerably promising for achieving a human-like presence to enhance human-machine interaction [42]. Consequently, the application of AI-based conversational avatars as digital human-like health carers has been gaining attention recently, leading patients to prefer digital human avatars as medical professionals over chatbots due to the former’s perceived human-like and interactive nature, which is essential for maintaining the professionalism and emotional aspects in physician-patient relationships [39].

Nonetheless, this evolution may apparently raise perceived uncanniness, which is considered a genuine ethical concern with realistic anthropomorphic avatars, yet it does not appear to be a universal problem for the avatar appearance or limit users’ engagement with the virtual environment [55]. Realistic humanoid avatars representing virtual carers can appear more trustworthy and increase compliance among users [55,109]. However, striking a balance between realistic and stylized avatar appearances can be worthwhile to avoid visual overloading and discomfort among users while representing the required elements accurately to maximize perceived trust, closeness, and acceptability [25].

#### Challenges and Limitations of CAs

The following challenges with CAs, including limitations in intelligence level, empathy, data privacy, and ensuring patient safety, highlight significant barriers to their adoption and effectiveness in health care.

##### Intelligence Level of CAs

Most of the proposed AI-based CAs were rule-based or scripted systems with a predetermined set of rules and conditions, which are easier to design and implement but have limited capability to handle complex conversations (unlike the automated ML-based systems) [33,56,118] and, therefore, cannot generate personalized responses in a more human-like manner, which the users would mostly prefer [84,91,108-110]. Furthermore, few studies proposed integrating personality-tailored behavior in CAs, precisely known as personality-adaptive CAs, which can automatically recognize and adapt to the users’ distinctive personality traits accordingly to be able to support patients in a personalized and authentic human-like manner [119,120] as well as effectively persuade users to alter their attitudes and behaviors for healthy lifestyle habits [121].

##### Empathy of CAs

Studies have emphasized the importance of including the “empathy” element in the design of CAs [122,123], yet only one of the included studies notably aimed to incorporate “empathy” in the proposed chatbot by applying text-based emotion analysis to recognize the user’s emotion and respond accordingly using emojis, stickers, and Graphics Interchange Format images [78]. Furthermore, a scoping review on AI-based CAs conducted by Kusal et al [56] has suggested that the major shortcoming of CAs is their lack of competence to make communication with humans seem natural with the aid of empathy and sentiments, mainly due to their failure to comprehend the human context and the users’ emotions as their responses are restricted to the queries they are trained for, which can lead to a frustrating user experience. Indeed, a study conducted by Casas et al [124] determined the capability of an empathetic chatbot to surpass a benchmark bot or even humans in predicting the emotional state of textual messages. Therefore, CAs should be able to model their context and select important information to remember as communication depends on past messages [56].

##### Data Privacy

Surprisingly, the included studies demonstrated limited emphasis on the data privacy of the AI-based CAs while handling patient data, which is a common challenge faced in the design and evaluation of CAs [111,125]. Hence, it is recommended to prioritize data privacy and confidentiality factors during the development of such technologies to gain users’ trust and acceptance [63].

##### Patient Safety

Another area that needs to be focused on is patient safety [126], which was also not highlighted in the included studies. For instance, the inability of digital health tools to recognize the urgency of the patient’s health condition may generate inappropriate health advice or lead to delayed treatment, increasing the risk of further complications. In addition, limited validation and medical training for existing CAs (as mentioned previously) poses risk of inaccuracy and raises concerns about patient safety. Therefore, assessing the validation status of these digital health tools is crucial for patient safety and optimal health care outcomes.

#### Uses and Benefits of AI-Based CAs in Managing NCDs

This review suggests that CAs are primarily used for supporting nonpharmacological interventions such as behavioral or lifestyle medication for managing NCDs such as CVDs, diabetes, obesity, cancer, and gastrointestinal diseases, as well as preventive measures for healthy living, indicating their potential ability to complementarily assist in the long-term remote management of NCDs [114,127,128]. The ubiquitous self-care support through constant symptom monitoring, along with the personalized behavioral modifications provided by CAs, can possibly rescue patients from minor health concerns and prevent the progression of chronic diseases [10,104]. Health care professionals are optimistic about CAs’ potential ability to save patients time and money that they usually spend on physical clinic visits [103], thereby reducing the health care cost and burden and increasing accessibility [91,104]. Augmented reality applications could enhance the training of health care professionals and even patients in using these new technologies effectively [115,129,130].

#### Efficacy

The efficacy evaluation of CAs for remote care is still not thoroughly explored and well understood. According to Monaco et al [63], inadequate evidence regarding the efficacy of digital health tools may have hindered the potential beneficial outcomes of using such resources to control NCDs. Nonetheless, the few included studies that aimed to identify the effectiveness of CAs in managing the target NCDs found favorable outcomes [10,24,82,90,96,97,105]. Moreover, many reviews have emphasized the efficacy of CAs in health care support. For instance, a systematic review conducted by Milne-Ives et al [26] investigated the effectiveness and usability of AI-based CAs designed for health care and found them generally effective, with positive or mixed evidence, whereas a narrative review by Dingler et al [60] used context-aware voice assistants (eg, Google Home and Amazon Echo), demonstrating the utility of speech-based CAs in providing personalized remote health care support. However, a systematic review conducted by Hossain et al [131] exhibited low positive outcomes of digital health interventions among people with NCDs in India, requiring further exploration of advanced technologies for the equitable and sustainable development of digital health tools.

#### Targeted Users

Most of the target user groups were based on chronic disease conditions rather than any specific age group. A fair proportion of studies (6/43, 14%) focused on the application of CAs in the older adult population, exhibiting that this vulnerable user group may be most in need of such technologies as older adults have greater risk of developing serious NCDs [28,58,61] and many older adult patients lead an isolated, lonely life and, therefore, require constant remote monitoring as well as a virtual companion for healthy living [132,133]. Hence, in other words, the selected user groups were mostly based on targeted chronic diseases that may be more prevalent in older adults. However, we did not encounter any significant study that modified the CAs to enhance accessibility for older adults, but social networking sites could potentially enhance engagement with such CAs among this user group [134].

Moreover, it is crucial to recognize that the vast majority (41/43, 95%) of the included studies were conducted in high-income countries, underscoring the necessity of further research among underserved populations, particularly in LMICs where individuals may be more vulnerable to developing NCDs due to limited health awareness and access to health care services.

### Limitations

Although this is an emerging field of research and we conducted an extensive literature search strategy, the possibility of noninclusion of relevant studies may still exist (eg, missing certain keywords or search terms, studies in languages other than English, studies not listed in the searched databases, and unidentified studies from gray literature). Similarly, our stringent inclusion and exclusion criteria were implemented to refine our focus on patient-centered AI-based CAs tailored for the remote management of NCDs, aiming to ensure the relevancy and applicability of our review to this particular domain of interest. However, this approach may have inadvertently narrowed the scope, potentially overlooking other relevant studies that could provide wider insights into our findings. Despite this, we strived to conduct a fair selection of studies meeting our inclusion criteria to mitigate selection bias and ensure a balanced representation of the literature. Nonetheless, it is important to acknowledge that other potential biases, such as publication bias, may still exist, which are beyond our control. In addition, new articles may have appeared after our search deadline (July 31, 2023), which may cause additional differentiations.

Furthermore, some common limitations were also identified within the included studies that may impact the overall findings of this review: (1) limited sample sizes (a vast majority of the studies had relatively small sample sizes, which could limit the generalizability of the findings), (2) short-term interventions or lack of long-term outcome measures (the duration of interventions in most studies was relatively short [80,82,88,101], potentially overlooking long-term user interactions and health outcomes essential for a comprehensive evaluation of CAs’ ability to sustainably assist in NCD management [10,37,77,85,91-93,96-98,103,109]), (3) limited demographic data (some studies lacked detailed demographic information of the participants [37,93,109], which may potentially limit the analysis of how demographic factors can impact the feasibility or effectiveness of the CAs), (4) potential bias (many studies had potential biases resulting from self-reported user data [37,77,78,101,109], sample bias [75,82,105], coding bias [108], and exclusion of variables or unaccounted confounding factors in analyses [100], which could impact the validity of the results), and (5) unaddressed technical aspects (some studies did not discuss the technical aspects of the proposed CAs, including their potential technical limitations or challenges [85,92,93]). Addressing these issues could contribute to the enhancement of the design, development, and usability of CAs in the future [135].

### Implications and Future Recommendations

As CAs strive to facilitate personalized and empathetic human-like conversations in the health care field [113], the necessity for context-oriented and domain-specific training emerges as a crucial consideration for improved accuracy and relevance [136]. Although advanced AI systems such as large language models trained on vast textual data (eg, the generative pretrained transformer series and LLaMA) have proven transformative in various sectors such as marketing, education, and customer service, their application in the health care sector remains underexplored, primarily due to a scarcity of relevant high-quality medical data sets [137]. In enhancing the AI-based CAs for health care, only a few recent studies have aimed to investigate real-world interactions between patients and health care professionals [136-139]. While recognizing the dynamic and nonstandardized nature of historical human interactions in health care, our recommendation to study real-world communications between patients and health caregivers stems from the objective of training CAs to be contextually relevant, particularly in addressing the unique challenges associated with specific chronic diseases. This approach may seek to empower CAs to navigate the complexities of diverse health conditions with greater intelligence and empathy, thereby improving their adaptability through the use of high-quality data sets acquired from real-world scenarios [136] given the substantial amount of data and extensive knowledge base required for domain-specific contextual training [56]. However, to ensure effective training of AI agents in health care, it is essential to define their roles (eg, nurse, health assistant, nutritionist, and pharmacist), which can determine the appropriate level of training required, allowing for the selection of specific data sets tailored to the medical training and evaluation of CAs.

Findings from the literature suggest an overall higher user preference for realistic human-like representations in CAs, specifically in the form of anthropomorphic digital avatars, which can be accessed through mobile devices (unlike physical humanoid robots) [52,54], thus accommodating a broad user base including older adult users [58,140]. Therefore, future research should prioritize investigating the impact of anthropomorphic avatars or virtual humans in managing NCDs, including their potential ethical concerns (eg, uncanny valley) that should be addressed in the design considerations to increase acceptance of CAs for self-care [25,49,123].

Moreover, user preferences related to CA types and features may vary notably among different user groups. For instance, older adults and users with disabilities may require voice-activated CAs over text-based CAs due to their typing inabilities, and therefore, enhanced speech recognition functionality is required to incorporate into CAs to accommodate voice conversations in multiple languages [70]. In addition to language requirements, factors such as culture, ethnicity, and individual personality traits may also play a significant role in influencing the adoption of AI dialogue systems for remote care purposes. Indeed, the adoption of CAs can be impeded among populations traditionally experiencing health inequities due to the limited availability of such tools with localized and culturally tailored features. These populations, often at higher risk of NCDs, could greatly benefit from more culturally sensitive health care technologies, which could improve health care access and outcomes, thereby necessitating careful consideration in future work.

### Conclusions

The review highlighted the promising acceptance of CAs by users for the self-management of chronic conditions, with feedback indicating helpfulness, satisfaction, and ease of use in most of the included studies. AI-based CAs present opportunities for communication and interaction in health care settings. However, understanding and optimizing the communication channels between humans and such CAs is crucial for enhancing their capabilities and potential benefits in health care. While our study confirmed the increasing role of CAs in augmenting self-care and potentially reducing health care costs, it also exposed critical limitations, particularly concerning conversational depth and emotional intelligence. This review also emphasized the lack of reliable and comparable evidence to determine the efficacy of AI-enabled CAs for chronic health conditions. Therefore, while user feedback and acceptance were positive, there is a need for more rigorous studies and standardized reporting to evaluate the effectiveness of AI-based CAs as human-like health carers for managing NCDs.

## Abbreviations

AI: artificial intelligence
CA: conversational agent
CVD: cardiovascular disease
LMIC: low- and middle-income country
ML: machine learning
NCD: noncommunicable disease
PRISMA-ScR: Preferred Reporting Items for Systematic Reviews and Meta-Analyses extension for Scoping Reviews

## Data Availability

The data sets generated during and analyzed during this study are available from the corresponding author on reasonable request.
